# Comparative Proteomic Analysis of *Puccinellia tenuiflora* Leaves under Na_2_CO_3_ Stress

**DOI:** 10.3390/ijms14011740

**Published:** 2013-01-15

**Authors:** Juanjuan Yu, Sixue Chen, Tai Wang, Guorong Sun, Shaojun Dai

**Affiliations:** 1 College of Life and Environmental Sciences, Shanghai Normal University, Shanghai 200234, China; E-Mail: yujuan8186@gmail.com; 2 Sanquan Medical College, Xinxiang Medical University, Xinxiang 453003, He’nan, China; 3 Department of Biology, Genetics Institute, Plant Molecular and Cellular Biology Program, Gainesville, FL 32610, USA; E-Mail: schen@ufl.edu; 4 Institute of Botany, Chinese Academy of Sciences, Beijing 100093, China; E-Mail: twang@ibcas.ac.cn; 5 Binzhou Polytechnic College, Binzhou 256603, Shandong, China; E-Mail: grsun@live.cn

**Keywords:** proteomics, halophyte, *Puccinellia tenuiflora*, Na_2_CO_3_ response

## Abstract

Soil salt-alkalinization is a widespread environmental stress that limits crop growth and agricultural productivity. The influence of soil alkalization caused by Na_2_CO_3_ on plants is more severe than that of soil salinization. Plants have evolved some unique mechanisms to cope with alkali stress; however, the plant alkaline-responsive signaling and molecular pathways are still unknown. In the present study, Na_2_CO_3_ responsive characteristics in leaves from 50-day-old seedlings of halophyte *Puccinellia tenuiflora* were investigated using physiological and proteomic approaches. Comparative proteomics revealed 43 differentially expressed proteins in *P. tenuiflora* leaves in response to Na_2_CO_3_ treatment for seven days. These proteins were mainly involved in photosynthesis, stress and defense, carbohydrate/energy metabolism, protein metabolism, signaling, membrane and transport. By integrating the changes of photosynthesis, ion contents, and stress-related enzyme activities, some unique Na_2_CO_3_ responsive mechanisms have been discovered in *P. tenuiflora*. This study provides new molecular information toward improving the alkali tolerance of cereals.

## 1. Introduction

Salt-alkali soil is one of the major abiotic constraints limiting crop distribution and yield worldwide [1–3]. Plant salinity tolerance has been extensively studied, but the understanding of plant alkalinity tolerance is lacking. The alkali salt affects plant growth and development through sodium toxicity and high pH, which are more likely to cause serious damage than neutral salt to the plant [4]. The high soil pH (mainly attributed to carbonate salts, e.g., Na_2_CO_3_ and NaHCO_3_) could directly affect nutrient uptake, organic acid balance, and ion homeostasis, especially the pH stability at cellular and whole plant levels [4,5]. Meanwhile, some halophyte species can naturally survive in high alkaline soil, and have evolved various regulatory and metabolic mechanisms for alkali tolerance.

Alkali grass (*Puccinellia tenuiflora*) is an alkali tolerant halophyte species that can survive in highly alkaline soil (e.g., pH 10). Thus, it is considered as an outstanding pasture for soil improvement. Although some salt tolerant mechanisms in *P. tenuiflora* have been studied before [6], few studies were focused on the specific molecular mechanisms underlying alkali tolerance. To cope with saline or alkaline stress, *P. tenuiflora* has developed various strategies, such as ion balance [7–9], osmotic adjustment [9–11], and reactive oxygen species (ROS) scavenging [12]. Previous studies have revealed that *P. tenuiflora* can remarkably accumulate citric acid in leaves and roots when exposed to alkaline stress. This is different from salt stress under which citric acid levels remain unchanged [9,11]. The accumulation of citric acid in *P. tenuiflora* may play an important role in pH adjustments used to cope with alkaline stress [9,11]. Besides, several ion salt-responsive genes encoding antiporters/channel proteins in *P. tenuiflora* have been isolated and transformed into yeast, rice and Arabidopsis to test their biological functions. These genes include *PutPMP3-1/2* [13], *PutHKT2;1* [14], *PutAKT1* [15], *KPutB1* [16], *PutCAX1* [17], and *PtNHA1* [18]. The specific functions of these genes have been summarized in our previous article [6]. Furthermore, some candidate salt/alkali-responsive genes/proteins in *P. tenuiflora* have been found using high-throughput transcriptomic and proteomic approaches. A cDNA library was constructed for *P. tenuiflora* under 450 mM NaHCO_3_ stress for 48 h. It contained a total of 95 differentially regulated transcripts [19]. Our previous comparative proteomic analysis revealed 93 unique NaCl-responsive proteins in *P. tenuiflora* leaves [6]. These studies have provided important information for understanding salt-tolerance mechanisms and candidate gene functions. However, the alkali responsive molecular processes remain elusive.

In the present study, we analyzed the characteristics of *P. tenuiflora* leaves in response to Na_2_CO_3_ using physiological and comparative proteomic approaches. By integrating the changes of photosynthesis, ROS scavenging enzymes activities, ion contents, and alkali-responsive proteins, some unique mechanisms of *P. tenuiflora* in response to Na_2_CO_3_ have been revealed, leading to better understanding of the underlying molecular mechanisms of alkali tolerance in cereals.

## 2. Results

### 2.1. Effects of Na_2_CO_3_ Stress on the Growth and Photosynthesis of P. tenuiflora

**Figure 1:**
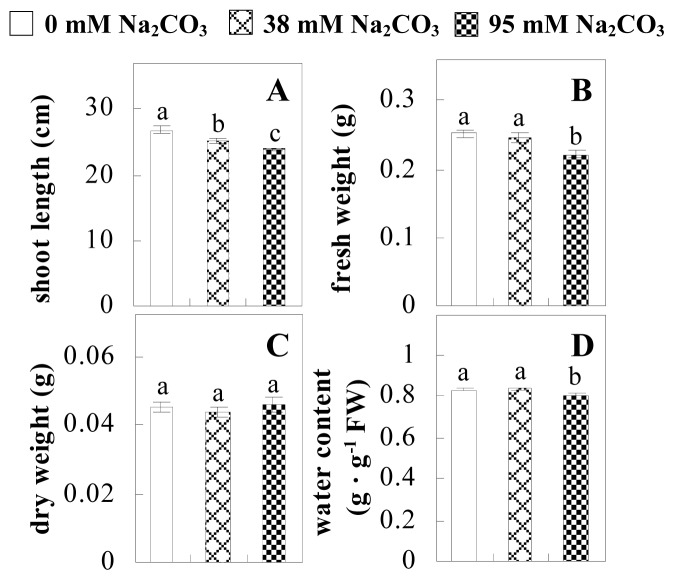
Biomass of *P. tenuiflora* seedlings grown under Na_2_CO_3_ conditions. (**A**) shoot length of seedlings; (**B**) fresh weight of leaves; (**C**) dry weight of leaves; (**D**) water content in leaves. The values were determined after plants were treated with 0 mM, 38 mM, and 95 mM Na_2_CO_3_ for seven days, and were presented as means ± SE (*n* = 9). The different small letters indicate significant differences (*p* < 0.05).

To evaluate the effects of alkaline stress on the growth of *P. tenuiflora*, shoot length, leaf fresh weight, dry weight, and water content were determined (Figure 1). The shoot length declined gradually with the increase in Na_2_CO_3_ concentration (Figure 1A). The fresh weight and water content decreased when seedlings were treated with 95 mM Na_2_CO_3_ (Figure 1B,D).

**Figure 2:**
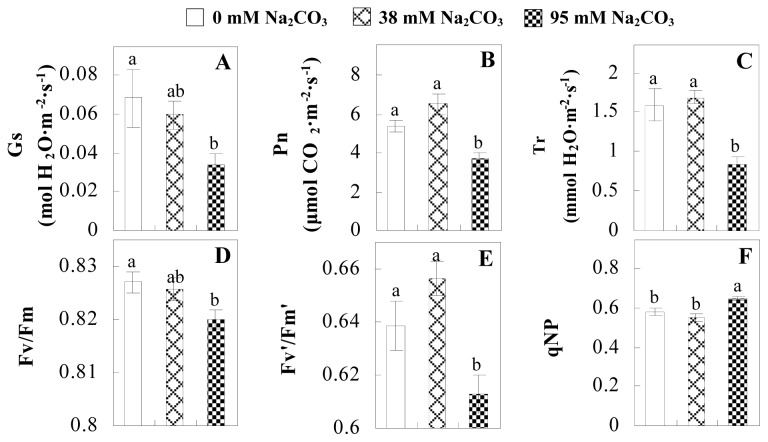
Photosynthetic characteristics (A, B, C) and chlorophyll fluorescence parameters (D, E, F) of *P. tenuiflora* leaves under Na_2_CO_3_ treatment. (**A**) stomata conductance (Gs); (**B**) photosynthesis rate (Pn); (**C**) transpiration rate (Tr); (**D**) Fv/Fm; (**E**) Fv′/Fm′; (**F**) qNP. The values were determined after plants were treated with 0 mM, 38 mM, and 95 mM Na_2_CO_3_ for seven days, and were presented as means ± SE (*n* = 9). The different small letters indicate significant differences (*p* < 0.05).

The photosynthesis indexes of *P. tenuiflora* under Na_2_CO_3_ treatment were analyzed. After seven days of 38 mM and 95 mM Na_2_CO_3_ treatments, the *P. tenuiflora* seedlings did not show obvious damage to leaf morphology (data not shown), implying the high capacity of *P. tenuiflora* seedlings to tolerate Na_2_CO_3_. However, photosynthesis was affected by Na_2_CO_3_ stress. Stomatal conductance (Gs) (Figure 2A), photosynthetic rate (Pn) (Figure 2B), and transpiration rate (Tr) (Figure 2C) exhibited little changes under 38 mM Na_2_CO_3_ treatment, but showed marked decreases under 95 mM Na_2_CO_3_. In addition, chlorophyll fluorescence parameters were monitored to determine the performance of photosystem II (PSII) photochemistry. The maximum quantum efficiency of PSII photochemistry (Fv/Fm) (Figure 2D) and the PSII maximum efficiency (Fv′/Fm′) (Figure 2E) were not significantly altered under 38 mM Na_2_CO_3_, but were reduced remarkably under 95 mM Na_2_CO_3_. The non-photochemical quenching coefficient (qNP) (Figure 2F) remained constant under 38 mM and then increased under 95 mM Na_2_CO_3_ treatment.

### 2.2. Changes to Leaf Osmotic Potential, Plasma Membrane Integrity and Antioxidant Enzyme Activities

**Figure 3:**
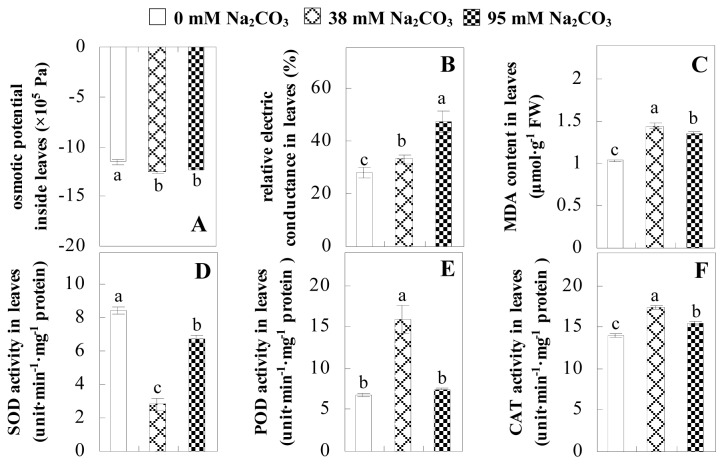
Changes of some antioxidant-related indexes in leaves of *P. tenuiflora* under Na_2_CO_3_ treatment. (**A**) osmotic potential; (**B**) electrolyte leakage ratio; (**C**) MDA contents; (**D**) SOD activity; (**E**) POD activity; (**F**) CAT activity. The values were determined after plants were treated with 0 mM, 38 mM, and 95 mM Na_2_CO_3_ for seven days, and were presented as means ± SE (*n* = 6). The small letters indicate significant difference (*p* < 0.05).

Leaf osmotic potential showed a significant decrease under Na_2_CO_3_ treatments (Figure 3A), indicating the seedlings suffered from osmotic stress. The electrolyte leakage ratio (Figure 3B) and malondialdehyde (MDA) contents (Figure 3C) were increased significantly under Na_2_CO_3_. This indicates that the plasma membrane integrity was damaged by Na_2_CO_3_ treatment, probably resulting from ROS generated under high pH and ion stress conditions. The activities of representative antioxidative enzymes were altered with different patterns under Na_2_CO_3_ stress. The superoxide dismutase (SOD) activity decreased under Na_2_CO_3_ stress (Figure 3D), but the peroxidase (POD) activity increased under 38 mM Na_2_CO_3_ (Figure 3E), and the catalase (CAT) activity increased obviously under both Na_2_CO_3_ concentrations (Figure 3F).

### 2.3. Ion Content Changes in Leaves under Na_2_CO_3_ Stress

**Figure 4:**
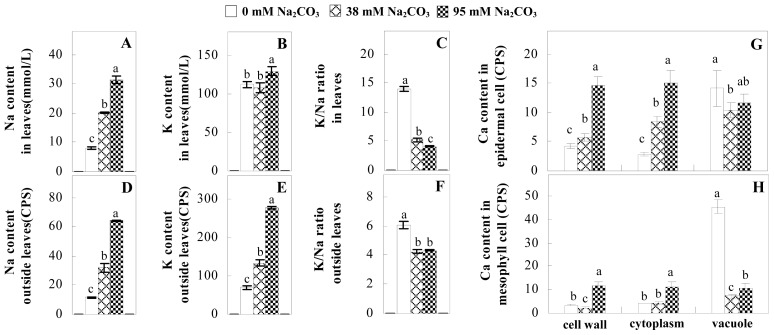
Changes in ionic contents in leaves of *P. tenuiflora* under Na_2_CO_3_ stress. (**A**) Na content in leaves; (**B**) K content in leaves; (**C**) K/Na ratio in leaves; (**D**) Na content outside leaves; (**E**) K content outside leaves; (**F**) K/Na ratio outside leaves; (**G**) Ca content in epidermal cells; (**H**) Ca content in mesophyll cells. The values were determined after plants were treated with 0 mM, 38 mM, and 95 mM Na_2_CO_3_ for seven days, and were presented as means ± SE (*n* = 6). The different letters indicate significant differences (*p* < 0.05). CPS, counts per second.

Ion homeostasis is important in plant response to salt stress. Leaf Na content increased with increases in Na_2_CO_3_ concentration (Figure 4A). Leaf K contents also increased under 95 mM Na_2_CO_3_ treatment (Figure 4B). This led to declined K/Na ratios under the Na_2_CO_3_ treatment (Figure 4C). Furthermore, Na and K contents on the leaf surface increased gradually with the increases in Na_2_CO_3_ concentration, and K/Na ratio was reduced on the leaf surface (Figure 4D,E,F). Moreover, as an important signaling messenger, calcium contents in different subcellular compartments of epidermal cells and mesophyll cells were affected by Na_2_CO_3_. In epidermal cells, calcium content in the cell wall and cytoplasm increased gradually with the increases in Na_2_CO_3_ concentration, but reduced in the vacuoles under 38 mM Na_2_CO_3_ treatment (Figure 4G). Similarly, in mesophyll cells, calcium contents in the cell wall and cytoplasm increased under 95 mM Na_2_CO_3_ treatments, while the calcium content in vacuoles reduced sharply in the Na_2_CO_3_ treated samples (Figure 4H).

### 2.4. Identification and Functional Categorization of Na_2_CO_3_ Responsive Proteins

**Figure 5:**
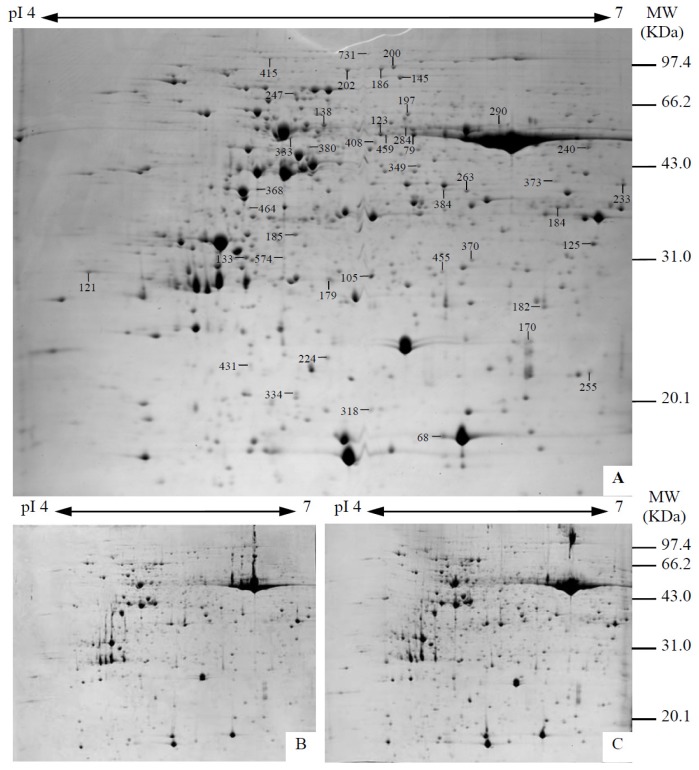
2-DE profiling of proteins extracted from *P. tenuiflora* leaves treated with different concentrations of Na_2_CO_3_. Gels A, B, and C are protein samples from leaves treated with 0, 38, and 95 mM Na_2_CO_3_ for seven days, respectively. Molecular weight (MW) in kilodaltons (kDa) and pI of proteins are indicated on the right and top of gel A and B, respectively. The 43 gel spots with protein IDs were marked with spot numbers. Detailed information can be found in Table 1.

The alkali-responsive protein profiles were obtained using 2-DE analysis of leaf samples under 0 mM, 38 mM, and 95 mM Na_2_CO_3_ treatments (Figure 5, Supplemental Figure S1). Approximately 1000 Coomassie Brilliant Blue-stained protein spots were detected on the pI 4–7 gels. A total of 43 protein spots were identified using LC ESI Q-TOF MS/MS and Mascot database searching (Figure 5, Table 1). Based on Gene Ontology, BLAST alignments, and information from literature, the protein identities (IDs) were classified into 10 functional categories including photosynthesis, stress and defense, membrane and transport, carbohydrate and energy metabolism, amino acid metabolism, transcription, protein synthesis, protein folding and transporting, protein degradation, and signaling (Table 1). Among these categories, carbohydrate and energy metabolism (41%), transcription and protein metabolism (27%), photosynthesis (14%), as well as stress and defense (9%), were over-represented.

### 2.5. Protein Clustering and the Dynamics of Protein Networks

**Figure 6:**
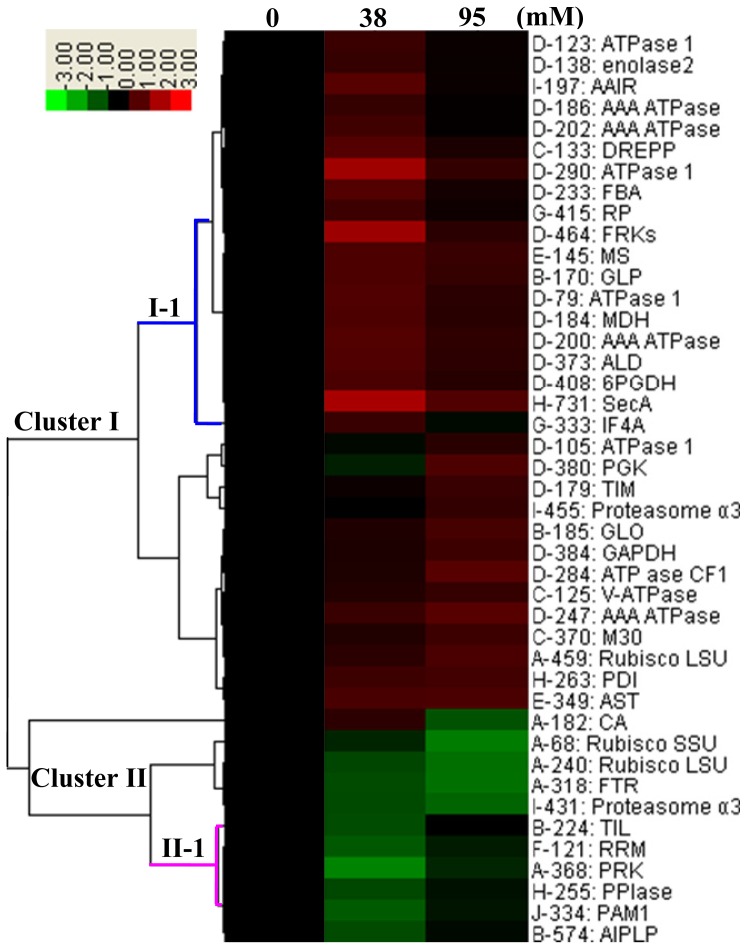
Hierarchical clustering analysis of the expression profiles of the identified 43 proteins. The three columns represent different treatments, *i.e.*, 0 mM, 38 mM, and 95 mM. The rows represent individual proteins. The protein cluster is on the left, and the treatment cluster is on the top. The increased and decreased protein spots were indicated in red and green, respectively. The intensities of the colors increase with the increase of expression differences, as shown in the bar on the top. The protein spot numbers are listed on the right, and the letters before the spot numbers represent various functional categories of the proteins. A, photosynthesis; B, stress and defense; C, membrane and transport; D, carbohydrate and energy metabolism; E, amino acid metabolism; F, transcription related; G, protein synthesis; H, protein folding and transporting; I, protein degradation; J, signaling.

One important goal of system biology is to understand the interdependence of proteins and their expression profiles in a certain tissue or other biological samples [6,20]. An effective method to determine the regulatory mechanisms for protein interactions is the application of hierarchical clustering algorithms used in DNA microarray experiments. With this method, the proteins appearing on the same branches are assumed to be involved in related molecular functions [6,20]. Thus, to analyze the expression characteristics of proteins involved in each functional category, we performed hierarchical clustering analysis of the 43 IDs, which revealed two main clusters. Cluster I included 32 alkali-induced IDs and cluster II contained 11 alkali-reduced IDs (Figure 6). The number of alkali-induced proteins was obviously larger than that of reduced proteins. The analysis of protein functional categories showed a heterogeneous distribution between the two clusters (Figure 6). For example, most of the IDs involved in carbohydrate and energy metabolism were grouped in cluster I, whereas photosynthesis-related proteins were mainly in cluster II (Figure 6). This suggests that a switch of biological processes occurred in the course of alkaline treatment. Interestingly, the clustering result supports the previous notion that 38 mM is the turning point concentration of Na_2_CO_3_ for *P. tenuiflora* [12,21,22], because there were 28 out of 43 protein IDs whose protein abundances changed. Among them, 22 proteins (sub-cluster I-1) reached the maximum levels, and six proteins (subcluster II-1) got to the minimum levels under 38 mM Na_2_CO_3_ treatment (Figure 6). Such protein patterns correlated well with our aforementioned physiological results, e.g., the photosynthetic capability (e.g., Pn, and qP) and antioxidant-related indexes.

## 3. Discussion

### 3.1. Photosynthesis Is Inhibited by Na_2_CO_3_

The effects of salinity and alkalinity on plant growth and development vary amongst plants. For glycophytes, salt stress generally reduces plant growth and development, but for most halophytes, moderate salt accumulation promotes the plant growth [23]. However, the less-tolerant dicotyledonous halophytes and monocotyledons halophytes, especially grasses, grow better in non-salinized conditions [23,24]. Our results showed that the growth of *P. tenuiflora*, a monocot halophyte grass, was not obviously affected by low Na_2_CO_3_ concentration, but was inhibited by higher Na_2_CO_3_ concentration (Figure 1). This correlates well with the biomass changes of *P. tenuiflora* under NaCl stress [6]. In this study, Gs, Pn, and Tr of *P. tenuiflora* seedlings exhibited little changes at 38 mM Na_2_CO_3_, but decreased significantly at 95 mM Na_2_CO_3_ (Figure 2A,B,C), indicating photosynthesis was reduced at the higher Na_2_CO_3_ concentration. Moreover, Fv/Fm and Fv′/Fm′ were stable at 38 mM Na_2_CO_3_, but reduced at 95 mM Na_2_CO_3_ (Figure 2D,E). This implied that the efficiency of PSII photochemistry was not affected by the low Na_2_CO_3_ concentration, but inhibited by the high Na_2_CO_3_ concentration. The reduced Fv/Fm of seedlings under 95 mM Na_2_CO_3_ implied the occurrence of photoinhibition in *P. tenuiflora* under the higher concentration of Na_2_CO_3_. The decrease of Fv/Fm was usually accompanied by increase of thermal dissipation, which was evaluated by nonphotochemical quenching of Chl fluorescence (qNP) [25]. Here qNP was maintained almost constant at 38 mM Na_2_CO_3_, but increased significantly at 95 mM Na_2_CO_3_ (Figure 2F), which corresponded with the change of Fv/Fm. This result implied that thermal dissipation remained steady at 38 mM Na_2_CO_3_, but increased at 95 mM Na_2_CO_3_.

Previous studies have found that salt and alkali stresses affected photosynthetic carbon fixation [1,26]. Our present proteomics data revealed that some of the enzymes in Calvin cycle were reduced by Na_2_CO_3_ stress, which implied that the decrease in Pn was due to the less efficient carbon fixation under Na_2_CO_3_ stress. These enzymes included carbonic anhydrase (CA), RuBisCO, phosphoribulokinase (PRK), and ferredoxin-thioredoxin reductase (FTR). CA can help increase the concentration of CO_2_ within the chloroplast in order to increase the carboxylation rate of RuBisCO [27]. The change tendency of CA is similar to those of Gs, Pn, and Tr in *P. tenuiflora*. These results indicate that the decrease of Pn is mainly resulted from the declined carbon fixation. RuBisCO catalyzes the first major step of carbon fixation in C3 plants, and PRK is the key enzyme that functions in phosphorylating RuP into ribulose-1,5-bisphosphate (RuBP) in the Calvin cycle [28]. FTR is an iron-sulfur enzyme, which links light to enzyme regulation in oxygenic photosynthesis, catalyzing the activation of fructose 1,6-bisphosphatase [29]. The decline of these enzymes in the Calvin cycle under Na_2_CO_3_ treatment could lead to the decrease of carbon fixation.

### 3.2. Antioxidant Mechanisms in Leaves to Cope with Na_2_CO_3_

Salt and alkali stresses enhance the production of ROS, resulting in various ROS-associated perturbations in the seedlings [2]. Chloroplasts are key intracellular ROS generators. In chloroplasts, the production of O_2_^−^ is mainly determined by the balance between absorption and utilization of light energy [25]. The energy consumption for CO_2_ assimilation suppressed by Na_2_CO_3_ stress led to ROS imbalance, which would cause oxidative damage to enzymes and thus the photosynthetic apparatus [2,25,30]. In the present study, the significantly increased electrolyte leakage ratio and MDA contents indicate that plasma membrane was damaged by lipid peroxidation under Na_2_CO_3_ stress [30].

Our results have revealed several mechanisms of light energy balance and antioxidation used to cope with Na_2_CO_3_ stress in *P. tenuiflora* seedlings. Salt accumulation on the leaf surface would prevent excessive light absorption in Na_2_CO_3_ stressed plants. The contents of Na and K on the surface of *P. tenuiflora* leaves increased gradually with increasing Na_2_CO_3_ concentrations (Figure 4A,B,D,E), which suggested that *P. tenuiflora* possesses the ability to secrete salts under Na_2_CO_3_ stress. Other ions such as Ca, Mg, and Si have also been found to increase in concentration on the *P. tenuiflora* leaf surface with the increasing Na_2_CO_3_ concentrations [10]. The accumulation of salts on leaf surfaces developed greater surface reflectance, contributing to reduce light absorption [31]. At the same time, thermal dissipation was enhanced to remove excess energy at high Na_2_CO_3_ concentration. The increase of qNP demonstrates that the thermal dissipation was enhanced to protect the photochemical apparatus under Na_2_CO_3_ stress (Figure 2F). In addition, the enhancement of the xanthophyll cycle would increase the thylakoid pH, which is helpful for induced thermal dissipation [25]. The increase of temperature-induced lipocalin (TIL) in our proteomics results supported this notion. Lipocalin was found to be a key enzyme in the xanthophyll cycle responsible for protection against photo-oxidative damage [32]. It was also found to be increased in *Solanum lycopersicum* under salt stress [33].

The ROS scavenging system was activated in seedlings to cope with Na_2_CO_3_ stress. The activities of POD (Figure 3E) and CAT (Figure 3F), the two enzymes involved in the removal of H_2_O_2_, were increased under Na_2_CO_3_ treatments, especially under 38 mM Na_2_CO_3_. However, SOD activity was reduced dramatically under Na_2_CO_3_ stress (Figure 3D). SOD is an enzyme for dismutation of O_2_^−^ to produce H_2_O_2_. Thus, in *P. tenuiflora* seedlings under Na_2_CO_3_, H_2_O_2_ might be mainly produced in peroxisome from oxidation of glycolate during photorespiration rather than from dismutation of O_2_^−^ [6]. In addition, our proteomics data showed that other antioxidant and detoxification mechanisms were enhanced in seedlings to cope with Na_2_CO_3_ stress. Glyoxalase (GLO) was found to be induced in seedlings with increasing concentrations of Na_2_CO_3_. GLO is a member of the glyoxalase system that carries out the detoxification of methylglyoxal and other reactive aldehydes produced in plant metabolism. Previous studies have documented that salinity stress induced high accumulation of methylglyoxal, a potent cytotoxin in various plant species [34]. In transgenic tobacco plants, overexpression of glyoxalase I can tolerate an increase in methylglyoxal and maintain high levels of reduced glutathione under salinity stress [35]. Our results implied that GLO might be an important candidate for conferring high alkali tolerance in *P. tenuiflora*. Furthermore, germin-like protein (GLP) was also induced under Na_2_CO_3_ stress. Germin was first detected in germinating wheat seeds, but its homologs have now become ubiquitous in the plant kingdom and have various functions, not only during embryogenesis, but also in biotic or abiotic stress conditions [36]. The increased GLPs were detected in *Nicotiana tabacum* leaf apoplast [37] and *Arabidopsis thaliana* roots [38] after exposure to salt stress. Wheat germin has been shown to display oxalate oxidase activity; this activity is shared among most plants [36]. Germin-like oxalate oxidase is involved in degrading the oxalic acid, a highly toxic chemical, through production of H_2_O_2_ [36]. Thus, the Na_2_CO_3_ increased GLP might provide an explanation for the decrease of oxalic acids in shoots and roots of *P. tenuiflora* [9]. Additionally, an aluminum-induced protein-like protein (AIPLP) was induced under 95 mM Na_2_CO_3_ treatment. Previous studies have shown that the AIPLP was not specific to aluminum stress, but also involved in other metal, wounding [39], and drought stresses [40]. This protein might also contribute to the tolerance to Na_2_CO_3_ stress in *P. tenuiflora* when at a high concentration.

### 3.3. Ion Homeostasis and Transport under Na_2_CO_3_ Stress

The intracellular ion homeostasis is fundamental to living cells. Under salinity conditions, high apoplastic levels of Na^+^ would alter the aqueous and ionic thermodynamic equilibrium, resulting in hyperosmotic stress, ionic imbalance, and toxicity [26]. Thus, proper regulation of ion flux is necessary for cells to keep the concentrations of toxic ions low and to accumulate essential ions. Our results implied that *P. tenuiflora* developed some protective mechanisms to reestablish cellular ion homeostasis through selective salt accumulation or exclusion, *in vivo* compartmentalization, and Ca^2+^ signaling.

Maintaining a high cytosolic K^+^/Na^+^ ratio is one of the most important mechanisms for plant salt tolerance [8]. In our study, the Na content in leaves increased remarkably in all alkaline treatments. However, the K content did not increase under 38 mM Na_2_CO_3_, but increased slightly under 95 mM Na_2_CO_3_ (Figure 4A,B). This led to the decreased intracellular K/Na ratio in *P. tenuiflora* leaves (Figure 4C), although some Na ions were secreted to the leaf surface (Figure 4D). It is obvious that the ion homeostasis in *P. tenuiflora* leaves was affected under Na_2_CO_3_ stress. In previous studies, *P. tenuiflora* had lower net Na^+^ uptake rates than wheat (less than 50% under 150 mM NaCl) [8], which indicates that *P. tenuiflora* has a greater capacity than wheat to restrict unidirectional Na^+^ influx to maintain low net Na^+^ uptake [9]. Besides, the increased contents of K and Na on the leaves surface with the increase of external Na_2_CO_3_ concentrations (Figure 4D,E) support the hypothesis that *P. tenuiflora* leaves could exude salts through stomata or together with wax secretion [10].

Ion compartmentalization in different tissues can facilitate their metabolic functions [2]. The salt-inducible Na^+^/H^+^ antiporter is in charge of Na removal from the cytoplasm or compartmentalization in the vacuoles [2]. The vacuolar Na^+^/H^+^ antiporters were induced by NaHCO_3_ in *P. tenuiflora*, suggesting its key role in pH regulation under alkaline conditions [41]. Vacuolar-type Na^+^/H^+^ antiporter was mainly driven by the proton gradient across the vacuolar membrane generated by vacuolar type H^+^-ATPases (V-ATPases) [2,42]. The V-ATPase is indispensable for plant growth under normal conditions due to its roles in energizing secondary transport, maintaining solute homeostasis, and facilitating vesicle fusion. Under stress conditions (e.g., salinity, drought, cold, acid, anoxia, and heavy metals), the survival of the cells depends strongly on maintaining or adjusting the activities of the V-ATPases [2,42]. In the present study, a subunit of V-ATPase was induced under Na_2_CO_3_ stress. The corresponding increase of Na content in vacuoles under Na_2_CO_3_ stress was much higher than in cytoplasm of epidermal cells and mesophyll cells [43]. These findings suggest that the Na_2_CO_3_-induced V-ATPase was required to energize the tonoplast for ion uptake into the vacuoles.

Importantly, calcium content was changed with diverse cellular structures (e.g., cell wall, cytoplasm, and vacuole) in epidermal cells and mesophyll cells in leaves of *P. tenuiflora* when exposed to Na_2_CO_3_ stress (Figure 4G,H). Calcium is a principal signaling molecule for salinity tolerance [2,3]. High salinity leads to increased cytosolic Ca^2+^, which initiates the stress signal transduction pathways [3]. In this study, the calcium content in cytoplasm of epidermal cells (Figure 4G) and mesophyll cells (Figure 4H) increased significantly under 95 mM Na_2_CO_3_. In contrast, the calcium content in vacuoles of epidermis cells (Figure 4G) and mesophyll cells (Figure 4H) decreased. This indicates that the increased cytosolic Ca^2+^ might be transported from the apoplast and intracellular compartments [2]. In addition, our proteomics data revealed that a developmentally regulated plasma membrane polypeptide (DREPP PM)-like protein increased under Na_2_CO_3_ stress. DREPP-like protein contains a possible Glu-rich site at the C terminus responsible for calcium binding. DREPP-like protein has been found to be increased temporarily in rice under cold acclimation [44] and salt stress [45]. This result suggests that DREPP-like protein may be associated with the Ca^2+^ signal transduction pathway in the seedlings of *P. tenuiflora* under Na_2_CO_3_ stress.

### 3.4. Enhancement of Energy Supply and Other Specialized Metabolism

In this study, nine protein IDs were carbohydrate metabolism-related enzymes, and nine were involved in energy production (Table 1). Among them, seven IDs (representing six unique proteins) were enzymes in glycolysis, including fructokinases (FRK), fructose-bisphosphate aldolase (FBA), triosephosphate isomerase (TIM), glyceraldehyde 3-phosphate dehydrogenase (GAPDH), phosphoglycerate kinase (PGK), and enolase. They were all induced under Na_2_CO_3_ stress. In fact, glycolysis shared a series of reversible reactions with gluconeogenesis. Interestingly, except for FRK, the remaining five enzymes catalyzing the reversible reactions were shared between glycolysis and gluconeogenesis. FRK, an enzyme that irreversibly catalyzes the transfer of a phosphate group from ATP to fructose in glycolysis, is the most important gateway in the control of sugar influx into glycolysis. Thus, the increase of FRK under Na_2_CO_3_ along with the other five enzymes would contribute to glucose breakdown for energy generation to cope with Na_2_CO_3_ stress. In addition, malate dehydrogenase (an enzyme in citric acid cycle) and cytosolic 6-phosphogluconate dehydrogenase (an enzyme involved in pentose phosphate pathway) were induced by Na_2_CO_3_ stress. Moreover, five homologs of ATP synthases and four proteins containing the AAA ATPase family protein domain were induced with similar patterns to aforementioned carbohydrate metabolism-related enzymes in leaves under Na_2_CO_3_ stress. This implies that the enhancement of the metabolism provides ATP for plant adaptation to Na_2_CO_3_ stress. Similar results have also been found in salt-stressed *Salicornia europaea* [20] and *P. tenuiflora* [6].

Two amino acid metabolism-related proteins, aspartate aminotransferase (AST) and methionine synthase (MS), increased in levels under Na_2_CO_3_ treatment (Table 1). AST catalyzes the interconversion of aspartate and α-ketoglutarate to oxaloacetate and glutamate, and plays an important role in nitrogen assimilation and biosynthesis of amino acids [20]. MS is an enzyme that catalyzes the final step in the regeneration of methionine from homocysteine [46]. l-methionine is the substrate for the synthesis of *S*-adenosyl-l-methionine (AdoMet), which is the major methyl-group donor for numerous transmethylations important in plants secondary metabolism. Lignin biosynthesis has been suggested to represent a major sink for AdoMet consumption in vascular plants. Thus, the increase of MS in Na_2_CO_3_-stressed *P. tenuiflora* could reflect an enhanced demand of AdoMet in the synthesis of lignin. In addition, AST and MS have been thought to be relevant to cell wall lignification [20], and a previous study has reported the increased deposition of lignin in the vascular tissues of plants under salinity stress [46]. Therefore, the increase of these two proteins might imply that Na_2_CO_3_ treatment can increase the vessel development in *P. tenuiflora.* The enhanced cell wall lignification increased the mechanical rigidity of cell wall, strengthening the vascular tissue and subsequently permitting water to be conducted through the xylem under negative pressure without collapse of the vessels. This structural alteration may enhance the cell-to-cell pathway for water transport, impart greater selectivity and reduced ion uptake, and compensate for diminished bulk flow of water and solutes along the apoplastic pathway [6,20,46].

Additionally, a number of proteins involved in transcription, protein synthesis, protein folding and transport, as well as protein degradation showed Na_2_CO_3_ response (Table 1). Among them, two Na_2_CO_3_-induced proteins were involved in protein folding. One was protein disulfide-isomerase (PDI), a member of a thioredoxin superfamily inserting disulfide bonds into folding proteins. PDI is specialized to accommodate the structural features of the membrane and secreted proteins. The other was a putative SecA, the mobile subunit of an integral membrane transporter in *Escherichia coli*, consuming ATP during the insertion and deinsertion phases of its catalytic cycle while guiding preprotein segments across the membrane [47]. While the exact function of SecA in plants exposed to akalinity stress is not known, its function is likely to affect membrane protein dynamics in *P. tenuiflora* under alkaline stress.

## 4. Experimental Section

### 4.1. Plant Cultivation and Treatment

Seeds of *Puccinellia tenuiflora* (Turcz.) scribn. et Merr. were sowed on pearlite and cultured in a controlled environment chamber under white fluorescent light (300 μM m^−2^ s^−1^; 13 h light/11 h dark) at 25 °C and about 75% relative humidity for 50 days, as previously described [6,12]. The plants were divided into three groups. In order to correlate with a number of our previous studies that used 0, 0.4%, 1.0% Na_2_CO_3_ treatments, these plants were treated with 0 mM, 38 mM and 95 mM Na_2_CO_3_, respectively. Three biological replicates were grown and treated with different concentrations. To maintain stable Na concentrations in Hoagland medium, we changed the culture medium daily and monitored the solution ion content and the osmotic potential using a SevenMulti Neutral meter (Mettler Toledo, London, UK) and a vapor pressure osmometer (5520, Wescor Inc., Logan, UT, USA), respectively. Seven days later, the treated and untreated leaves were harvested and used fresh or immediately frozen in liquid nitrogen and stored at −80 °C for physiological and proteomic analysis. Shoot length and fresh weight were measured right after harvesting. Dry weight was determined after dehydration at 60 °C until a constant weight was maintained. Leaf water content was estimated as the difference between the fresh weight and dry weight divided by the fresh weight [6].

### 4.2. Photosynthesis and Chlorophyll Fluorescence Analysis

Net photosynthetic rate (Pn), stomatal conductance (Gs), and transpiration rate (Tr) were determined at 10:00 am using a portable photosynthesis system LI-COR 6400 (LI-COR Inc., Lincoln, NE, USA). The induction kinetics of chlorophyll fluorescence was recorded at room temperature using a pulse modulation chlorophyll fluorometer (FMS-2, Hansatech, King’s Lynn, UK). After dark adaptation for 30 min, the initial fluorescence yield (Fo) in weak modulated light (0.12 μmol photons m^−1^ s^−1^, 600 Hz), and maximum fluorescence yield (Fm) emitted during a saturating light pulse (5000 μmol photons m^−2^ s^−1^, 0.7 s) were measured. Variable fluorescence (Fv) was calculated by the formula: Fv = Fm−Fo. Then Fm′ was measured using an irradiance of 400 μmol photons m^−2^ s^−1^ as the actinic light and 5000 μmol photons m^−2^ s^−1^ as the saturating flashes. Fo′ was measured using an irradiance of far-red light (1.67 μmol photons m^−2^ s^−1^, 3 s). The variable fluorescence (Fv′) was also calculated by Fv′ = Fm′−Fo′. Maximal photochemical efficiency of PSII (Fv/Fm), photochemical efficiency of PSII in the light (Fv′/Fm′), non-photochemical quenching coefficient (qNP; 1−(Fm′−Fo′)/(Fm−Fo)), could be acquired directly from the instrument [12].

### 4.3. Analysis of Osmotic Potential, Electrolyte Leakage, MDA Content, and Antioxidant Enzymes

Osmotic potential was determined by a vapor pressure osmometer (5520, Wescor Inc., Logan, UT, USA). The MDA content was determined using the thiobarbituric acid (TBA) reaction as described by Wang *et al.* [48]. The electrolyte leakage ratio was obtained according to Akram *et al.* [49]. The three antioxidant enzymes (SOD, POD, and CAT) were extracted and detected according to the method of Yu *et al.* [6].

### 4.4. Determination of Ion Content

For measuring the relative contents of K and Na on the leaf surface, fresh leaves (1 × 2 cm) were cemented to a sample plate, then observed and analyzed with XL-30 surrounding scanning electron microscope (The Netherlands Phillips Company, Eindhoven, Netherlands) and Kevex energy peatrometer (Thermo Company, Schaumburg, IL, USA). The working voltage was 20 kV, and the time difference for the measurement of samples was 60 s. The relative contents of elements were represented as CPS (counts per second), which were computed by subtracting the background value from peak values of varied elements.

The sample preparation and microscopic analysis for Ca determination was done according to Qi *et al.* [50]. The relative Ca contents in diverse cellular structures (including cell wall, cytoplasm, vacuole) were detected by transmission electron microscope (TEM)-X ray microanalysis of 1 μM sections after freeze-drying and water-free embedding in plastic. The leaves (2 mm × 2 mm) were placed in aluminum mesh boxes, and then frozen quickly in the pre-cooling isopentane and propane mixture (the volume ratio is 1:3). Once frozen, the material was placed in a freeze-dryer for removing all of the water. Dry materials were then loaded into the T-type vacuum permeable tube, and infiltrated with diethyl ether in vacuum at 27 °C for 24 h. After being infiltrated with phenylethylene-butyl methacrylate under normal temperature, the materials were transferred into capsules for polymerization. The embedding materials were cut into 1 μm thickness sections on an ultramicrotome (Reichert Ultracut E, Vienna, Austria). For X-ray microanalysis, dry sections were transferred to folding copper grids, coated with carbon and stored over silica gel. Sections were analyzed in a Hitachi 800 transmission electron microscope (fitted with an energy-dispersive X-ray analyzer EDAX 9100). The accelerating voltage was 150 kV, the angle between sample and probe was 33°, and the counting time was 60 s. The relative Ca contents in diverse cellular structures (including cell wall, cytoplasm, vacuole) were also represented by CPS.

For measuring the concentrations of K^+^ and Na^+^ in the seedling leaves, 0.5 g fresh leaves of each treatment were cut to 2 cm, and put into a small beaker, then immersed in 30 mL deionized water and shaken for 30 min. The leaves were taken out and put into another small beaker with about 30 mL deionized water, then cooked for 15 min in the autoclave with high temperature (121 °C) in order to kill the cells and release all the ions. Finally, after the temperature of the solution was cooled to room temperature, the leaves were filtered from the solution, and the extracted solution was added to the volume of 50 mL. We used the SevenMulti tester to determine the concentrations of Na^+^ and K^+^.

### 4.5. Protein Sample Preparation, 2DE, and Image Analysis

The proteins from leaves under different treatment conditions were extracted according to the method of Wang *et al.* [48]. Protein samples were prepared independently from three different batches of plants. Protein concentration was determined using a Quant-kit according to manufacture’s instructions (GE Healthcare, Piscataway, NJ, USA). The protein samples were separated and visualized using 2DE approaches according to Yu *et al.* [6]. Protein samples were separated on 24 cm IPG strips (pH 4–7 linear gradient) through isoelectric focusing (IEF) in the first dimension, followed by 12.5% SDS-PAGE gels in the second dimension. About 1.6 mg protein was loaded per gel. Gels were stained by Coomassie Brilliant Blue (CBB). Three biological replicates for each sample and three technical replicates for each biological replicate were run on 2D gels. Gel image acquisition and analysis were conducted as previously described [48]. For image acquisition, the gels were scanned using an ImageScanner III (GE Healthcare) at a resolution of 300 dpi and 16-bit grayscale pixel depth. The images were analyzed with ImageMaster 2D software (version 5.0) (GE Healthcare, Piscataway, NJ, USA).The average vol% values were calculated from three technical replicates to represent the final vol% values of each biological replicate. Spots with more than 1.5-fold change among the treatments and a *p* value smaller than 0.05 were considered to be differentially expressed.

### 4.6. Protein Identification and Database Searching

The differentially expressed spots were excised from the gels and digested with trypsin [51]. MS/MS spectra were acquired on a LC ESI Q-TOF MS/MS (AB Sciex, Framingham, MA, USA) according to the method of Yu *et al.* [6]. The MS/MS spectra were searched against the NCBInr protein databases [52] using Mascot software (Matrix Sciences, London, UK). The taxonomic category was green plants. The searching criteria were according to Yu *et al.* [6].

### 4.7. Protein Classification and Hierarchical Cluster Analysis

The identified proteins were searched against the NCBI database [52] and UniProt database [53] to determine if their functions were known. Combined with the result of BLAST alignments, these proteins were classified into different categories based on biochemical functions. Self-organizing tree algorithm hierarchical clustering of the expression profiles was performed on the log transformed fold change values of protein spots [20,54].

### 4.8. Statistical Analysis

All results were presented as means ± standard error (SE) of at least three replicates. Data were analyzed by one-way ANOVA using the statistical software SPSS 17.0 (SPSS Inc., Chicago, IL, USA). The treatment mean values were compared by post hoc least significant difference (LSD) test. A *p* value less than 0.05 was considered statistically significant.

## 5. Conclusion

Current proteomics allows detailed inspection of individual proteins on a large scale and their dynamic changes underlying different alkaline-responsive cellular processes. In the present study, we discovered several Na_2_CO_3_-induced enzymes, such as xanthophyll cycle-related TIL, ROS scavenging enzymes (e.g., GLO and GLP), enzymes involved in carbohydrate metabolism, and amino acid metabolism-related proteins (e.g., AST and MS). A number of proteins involved in carbon assimilation, transcription, as well as proteins synthesis and fate were found to be reduced under Na_2_CO_3_ treatment. In addition, several proteins were proposed to be involved in cross-tolerance (e.g., AIPLP and DREPP-like protein). More than half of the Na_2_CO_3_-responsive proteins reached the maximum or minimum abundances under 38 mM Na_2_CO_3_ treatment. This correlates to the performance of plant growth and physiological indexes (e.g., photosynthetic capability and antioxidant enzyme activities), which supports the previous notion that 38 mM Na_2_CO_3_ is the turning point concentration for *P. tenuiflora.* Based on the integration of proteomics and physiological results, our study revealed several Na_2_CO_3_-responsive mechanisms in *P. tenuiflora*, including declined photosynthesis (e.g., light absorption and carbon assimilation) and activation of multiple antioxidant mechanisms (e.g., salt ion exclusion and compartmentalization; POD pathway, CAT pathway, and glyoxalase system), as well as enhanced energy supply and other specialized metabolisms. All these findings provide useful molecular information toward improving alkali tolerance of cereals.

## Figures and Tables

**Table 1:** Proteins and their relative changes in leaves from *P.tenuiflora* under Na_2_CO_3_ treatment.

Spot No. a	Protein name b	Plant species c	gi Number d	Thr. MW (Da)/pI e	Exp. MW (Da)/pI f	Sco g	Cov (%) h	QM i	V% ± SE j 0, 38, 95 mM Na_2_CO_3_
	**Photosynthesis (6)**								
182	carbonic anhydrase, chloroplast precursor (CA)	*Hordeum vulgare*	729003	35,736/8.93	30,029/6.47	125	6	2	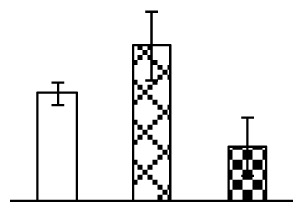
459	Rubisco large subunit (Rubisco LSU)	*Tristachya leucothrix*	125991685	51,294/6.23	64,412/5.71	86	19	8	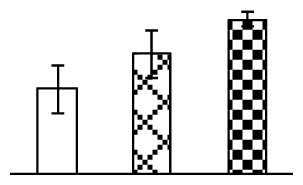
240	Rubisco large subunit (Rubisco LSU)	*Orobanche coerulescens*	46410750	47,410/6.45	62,047/6.68	176	10	4	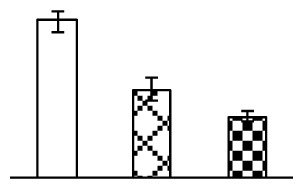
68	Rubisco small subunit (Rubisco SSU)	*Avena sterilis subsp. ludoviciana*	3790104	19,030/8.29	4088/5.98	78	9	2	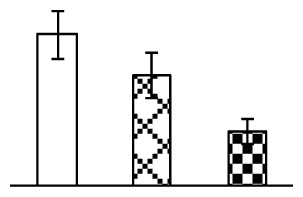
368	phosphoribulokinase (PRK), chloroplast precursor	*Oryza sativa*	125578	44,486	53,645/5.08	210	14	6	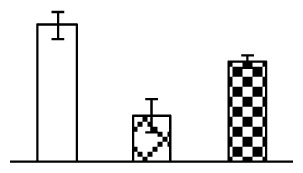
318	ferredoxin-thioredoxin reductase, variable chain (FTR)	*Zea mays*	2498397	10,937/5.69	9350/5.63	49	14	2	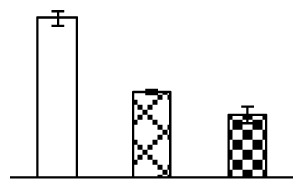
	**Stress and defense (4)**								
224	temperature stress-induced lipocalin (TIL)	*Triticum aestivum*	18650668	21,809/5.50	19,791/5.42	114	7	1	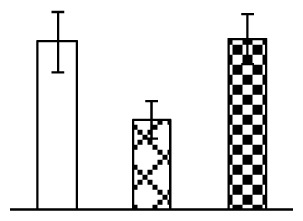
185	glyoxalase I (GLO)	*O. sativa (japonica cultivar-group)*	16580747	32,861/5.51	44,631/5.27	202	15	6	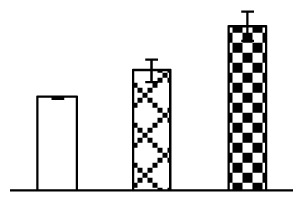
170	germin-like protein 1 (GLP)	*O. sativa*	4239821	22,017/6.01	22,973/6.40	84	10	1	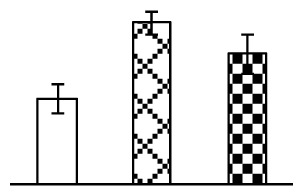
574	aluminum-induced protein-like protein (AIPLP)	*Setaria italica*	124263781	27,004/6.05	39,899/5.21	114	9	2	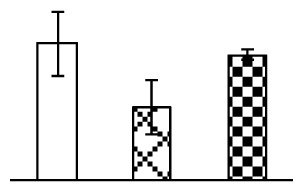
	**Membrane and transport (3)**								
133	Os01g0233000, containing pfam05558 DREPP plasma membrane polypeptide domain (DREPP)	*O.sativa (japonica cultivar-group)*	115435500	21,788/4.92	40,022/4.03	89	7	2	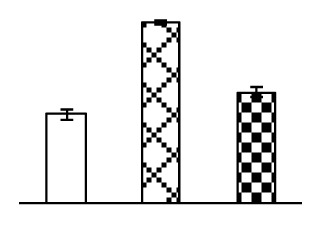
125	Vacuolar-type H^+^-ATPase (V-ATPase)	*H. vulgare*	2493132	53,806/5.12	42,714/6.71	70	3	2	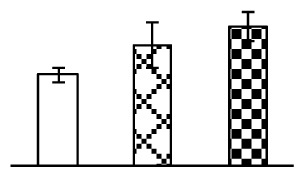
370	membrane-associated 30 kDa protein, chloroplast precursor (M30)	*Pisum sativum*	729842	35,709/9.30	39,410/6.12	80	6	2	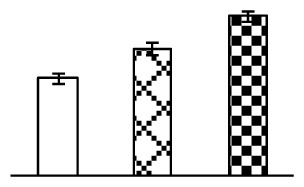
	**Carbohydrate and energy metabolism (18)**								
464	Os08g0113100, containing cd01167 fructokinases (FRKs) domain	*O. sativa (japonica cultivar-group)*	115474481	35,893/5.02	49,892/5.04	127	5	1	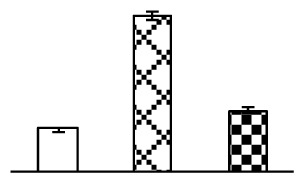
233	putative fructose-bisphosphate aldolase (FBA)	*Phleum pratense*	5419990	25,028/7.79	54,664/6.85	132	17	2	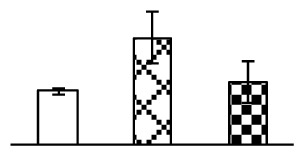
373	cytoplasmic aldolase (ALD)	*O. sativa*	218157	39,151/6.56	55,317/6.53	226	10	7	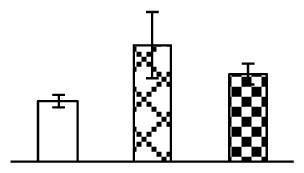
179	triosephosphate isomerase, cytosolic (TIM)	*Secale cereale*	1174749	27,138/5.24	35,657/5.43	79	13	3	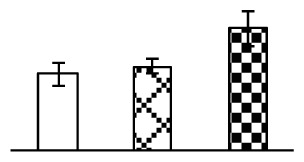
384	Os03g0129300, containing pfam00044 Gp_dh_N (GAPDH) domain	*O. sativa (japonica cultivar-group)*	115450493	47,537/6.22	53,685/5.98	239	12	8	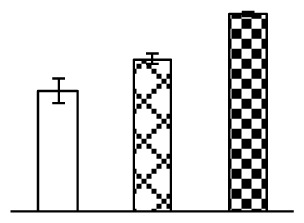
380	phosphoglycerate kinase (PGK)	*Vitis vinifera*	147843754	42,510/6.29	62,210/5.33	137	9	3	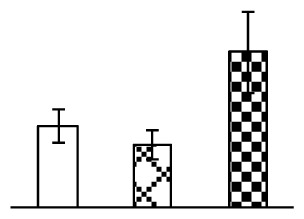
138	enolase2	*Z. mays*	162460735	48,418/5.70	66,697/5.4	143	10	3	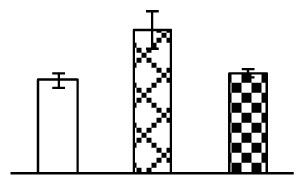
184	malate dehydrogenase, mitochondrial precursor (MDH)	*Citrullus lanatus var. lanatus*	126896	36,406/9.68	50,177/6.54	78	3	2	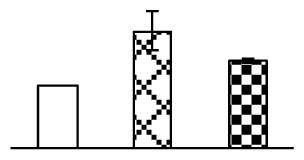
408	putative cytosolic 6-phosphogluconate dehydrogenase (6PGDH)	*Z. mays*	3342802	53,204/6.24	63,107/5.65	64	4	2	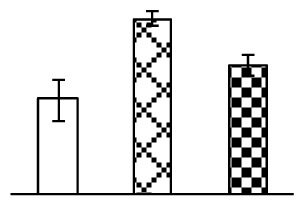
79	ATP synthase alpha (ATPase 1)	*T. aestivum*	81176509	55,557/5.70	64,698/5.84	361	12	4	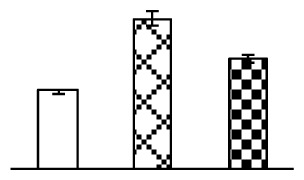
123	ATP synthase alpha subunit (ATPase 1)	*T. aestivum*	81176509	55,557/5.70	64,820/5.68	207	39	19	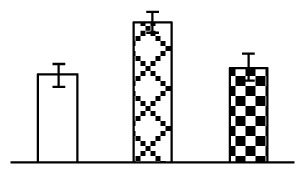
290	ATP synthase alpha subunit (ATPase 1)	*Elymus sibiricus*	51556908	55,549/6.03	66,615/6.26	77	20	8	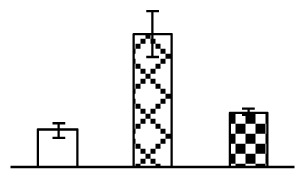
284	ATP synthase CF1 alpha subunit (ATP ase CF1)	*Agrostis stolonifera*	118430299	55,491/6.11	66,044/5.8	88	24	9	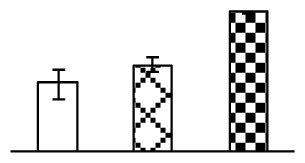
105	mitochondrial ATP synthase precursor (ATPase 1)	*T. aestivum*	47607439	27,090/7.71	36,228/5.63	62	6	2	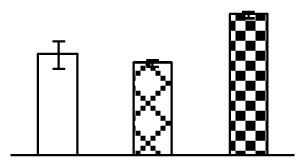
200	Os12g0230100, containing two AAA ATPase family protein domain	*O. sativa (japonica cultivar-group)*	115487910	102,068/6.62	78,280/5.74	87	16	13	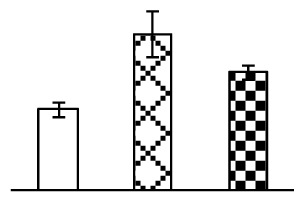
202	hypothetical protein OsI_036614, containing two AAA ATPase family protein domain	*O. sativa (indica cultivar-group)*	125536167	156,826/7.37	77,750/5.52	73	4	10	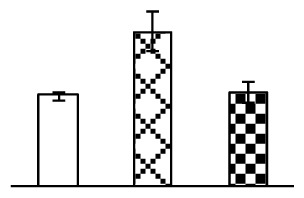
186	unnamed protein product, containing cd00009 the AAA, ATPases domain	*Vitis vinifera*	157343871	50,950/5.8	78,076/5.68	146	37	16	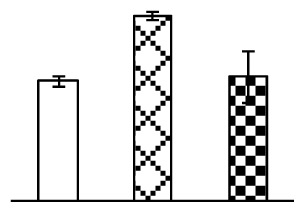
247	hypothetical protein OsI_023646, containing AAA, ATPases domain	*O. sativa (indica cultivar-group)*	125556808	64,173/5.65	72,936/5.27	74	22	12	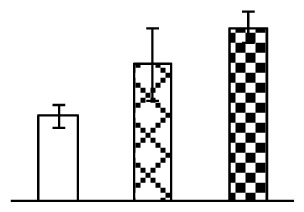
	**Amino acid metabolism (2)**								
349	aspartate aminotransferase (AST)	*O. sativa*	29468084	46,016/5.90	58,376/5.86	375	16	7	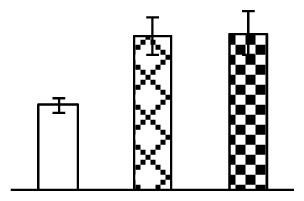
145	methionine synthase (MS)	*H. vulgare subsp. vulgare*	50897038	84,794/5.68	76,241/5.77	112	4	3	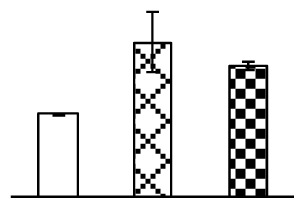
	**Transcription related (1)**								
121	cp31BHv, containing cd00590 RNA recognition motif (RRM) domain	*H. vulgare subsp. vulgare*	3550483	30,662/4.76	37,167/4.27	115	8	2	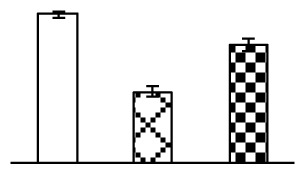
	**Protein synthesis (2)**								
333	eukaryotic initiation factor 4A (IF4A)	*O. sativa (japonica cultivar-group)*	303844	47,187/5.29	64,045/5.24	166	12	3	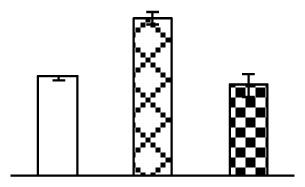
415	Os07g0168000, containing cd00164 Ribosomal protein S1-like RNA-binding domain(RP)	*O. sativa (japonica cultivar-group)*	115470767	98,023/5.64	79,993/5.15	84	3	2	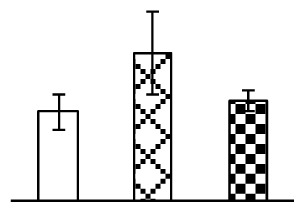
	**Protein folding and transporting (3)**								
255	peptidyl-prolyl cis-trans isomerase, chloroplast precursor (PPIase)	*Glycine max*	9899901	18,841/8.49	17,018/6.69	146	19	2	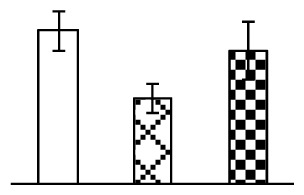
263	protein disulfide-isomerase precursor (PDI)	*Nicotiana tabacum*	1848212	40,082/5.99	53,400/6.09	92	6	3	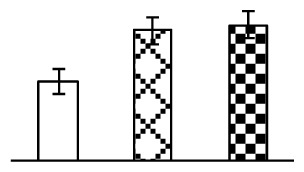
731	putative SecA	*O. sativa (japonica cultivar-group)*	52075758	114,899/5.78	80,931/5.62	88	11	10	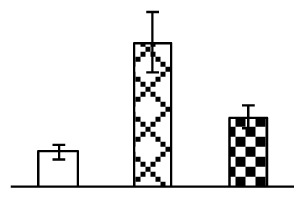
	**Protein degradation (3)**								
455	Os01g0811100, containing cd03751 proteasome alpha type 3 (Proteasome α3) domain	*O. sativa (japonica cultivar-group)*	115440617	27,506/5.75	37,044/5.97	107	6	2	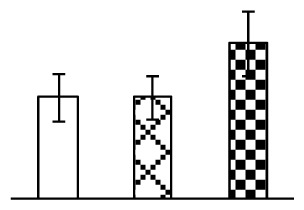
431	unknown, containing cd03751 proteasome alpha type 3 (Proteasome α3) domain	*H. vulgare*	117670154	27,448/5.82	18,201/5.05	167	15	4	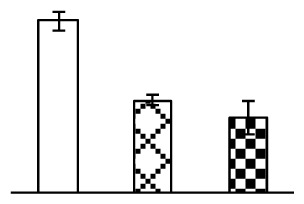
197	Os05g0573700, containing pfam07991 acetohydroxy acid isomeroreductase, catalytic domain (AAIR)	*O. sativa (japonica cultivar-group)*	115465569	62,680/6.01	68,899/5.81	254	11	6	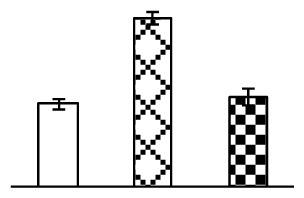
	**Signaling (1)**								
334	plant adhesion molecule PAM1	*Arabidopsis thaliana*	22531279	34,306/8.80	12,776/5.27	74	17	6	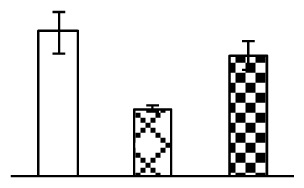

a Assigned spot number as indicated in Figure 5;

b The name and functional categories of the proteins identified using LC ESI Q-TOF MS/MS;

c The plant species that the peptides matched to;

d Database accession numbers from NCBInr;

e, f Theoretical (e) and experimental (f) mass (kDa) and pI of identified proteins. Experimental values were calculated using Image Master 2D Platinum Software. Theoretical values were retrieved from the protein database;

g The amino acid sequence coverage for the identified proteins;

h The Mascot score obtained after searching against the NCBInr database;

i The number of unique peptides identified for each protein;

j The mean values of protein spot volumes relative to total volume of all the spots. Three Na_2_CO_3_ treatments (0 mM, 38 mM, 95 mM) were performed. Error bars indicate ± standard error (SE).

## Abbreviation

6PGDH: 6-phosphogluconate dehydrogenase
AAIR: acetohydroxy acid isomeroreductase
AIPLP: aluminum-induced protein-like protein
ALD: aldolase
AST: aspartate aminotransferase
ATP ase CF1: ATP synthase CF1 alpha subunit
ATPase 1: ATP synthase alpha subunit
CA: carbonic anhydrase
CAT: catalase
CPS: counts per second
DREPP: developmentally regulated plasma membrane polypeptide
FBA: fructose-bisphosphate aldolase
FRKs: fructokinases
FTR: ferredoxin-thioredoxin reductase
Fv/Fm: maximum quantum efficiency of PSII photochemistry
Fv′/Fm′: PSII maximum efficiency
GAPDH: glyceraldehyde-3-phosphate dehydrogenase
GLO: glyoxalase
GLP: germin-like protein 1
Gs: Stomatal conductance
IDs: identities
IF4A: eukaryotic initiation factor 4A
M30: membrane-associated 30 kDa protein
MDA: malondialdehyde
MDH: malate dehydrogenase
MS: methionine synthase
PDI: protein disulfide-isomerase
PGK: phosphoglycerate kinase
Pn: photosynthetic rate
POD: peroxidase
PPIase: peptidyl-prolyl cis-trans isomerase
PRK: phosphoribulokinase
Proteasome α3: proteasome alpha type 3
PSII: photosystem II
qNP: non-photochemical quenching coefficient
ROS: reactive oxygen species
RP: Ribosomal protein S1-like RNA-binding domain
RRM: RNA recognition motif
Rubisco LSU: Rubisco large subunit
Rubisco SSU: Rubisco small subunit
SOD: superoxide dismutase
TIL: temperature stress-induced lipocalin
TIM: triosephosphate isomerase
Tr: transpiration rate
V-ATPase: vacuolar-type H; ^+^-ATPase
